# Endothelial EphrinB2 Regulates Sunitinib Therapy Response in Murine Glioma

**DOI:** 10.3390/life12050691

**Published:** 2022-05-06

**Authors:** Thomas Broggini, Lena Stange, Kristin Elizabeth Lucia, Peter Vajkoczy, Marcus Czabanka

**Affiliations:** 1 Department of Neurosurgery, University Hospital Frankfurt, 60528 Frankfurt am Main, Germany; lena.stange@kgu.de (L.S.); kristin.lucia@kgu.de (K.E.L.); marcus.czabanka@kgu.de (M.C.); 2 Department of Neurosurgery, Charité-Universitätsmedizin Berlin, 10117 Berlin, Germany; peter.vajkoczy@charite.de

**Keywords:** glioma, glioblastoma, antiangiogenic therapy, ephrinB2, EphB4

## Abstract

Vascular guidance is critical in developmental vasculogenesis and pathological angiogenesis. Brain tumors are strongly vascularized, and antiangiogenic therapy was anticipated to exhibit a strong anti-tumor effect in this tumor type. However, vascular endothelial growth factor A (VEGFA) specific inhibition had no significant impact in clinical practice of gliomas. More research is needed to understand the failure of this therapeutic approach. EphrinB2 has been found to directly interact with vascular endothelial growth factor receptor 2 (VEGFR2) and regulate its activity. Here we analyzed the expression of ephrinB2 and EphB4 in human glioma, we observed vascular localization of ephrinB2 in physiology and pathology and found a significant survival reduction in patients with elevated ephrinB2 tumor expression. Induced endothelial specific depletion of ephrinB2 in the adult mouse (*efnb2*^i∆EC^) had no effect on the quiescent vascular system of the brain. However, we found glioma growth and perfusion altered in *efnb2*^i∆EC^ animals similar to the effects observed with antiangiogenic therapy. No additional anti-tumor effect was observed in *efnb2*^i∆EC^ animals treated with antiangiogenic therapy. Our data indicate that ephrinB2 and VEGFR2 converge on the same pathway and intervention with either molecule results in a reduction in angiogenesis.

## 1. Introduction

Gliomas are brain tumors derived from brain glia cells and represent 80% of primary brain malignancies [1]. Treatments are slowly advancing, and primary care consists of maximal surgical resection in combination with radiation and chemotherapy [2,3]. Gliomas are strongly vascularized and show infiltrative behavior along blood vessels [4]. Therefore, anti-vascular therapy was hypothesized to be highly effective in these tumors [5]. The high expectations for antiangiogenic therapy in glioma were unable to be upheld as shown by evidence from multiple clinical trials in which treatment resistance was highly abundant [6,7,8,9,10,11]. As such, further research is needed to identify mechanisms of resistance. Preclinical data have identified the involvement of the cell—cell interaction surface receptor EphB4 [12,13]. EphB4—ephrinB2, receptor–ligand interaction is essential during development as the genetic elimination of either gene results in embryonic lethality [14,15,16]. Moreover, ephrinB2 was identified to play a critical role in the regulation of VEGFR2 signaling during vasculogenesis and angiogenesis [17]. The ephrinB2 PDZ domain is required to control VEGFR2–ephrinB2 dimerization and internalization in endothelial cells [17]. Hence, ephrinB2 regulates VEGFR2′s main downstream signaling pathway. These findings indicate that antiangiogenic therapy and ephrinB2 blockage converge on the same downstream pathways. We therefore hypothesized that genetic ephrinB2 depletion and antiangiogenic therapy result in similar phenotypes. Additionally, we aimed to research additive anti-tumor effects of genetic ephrinB2 depletion and antiangiogenic therapy.

## 2. Materials and Methods

### 2.1. In-Silico Data Collection and Analysis

The expression data on physiological expression in human and mouse development were downloaded from the Allen Institute, accessed on 4 April 2022 (https://portal.brain-map.org/) [18,19]. The pathological ephrinB2/EphB4 histology was received from the Human Protein Atlas, accessed on 15 March 2022 (https://www.proteinatlas.org/) [20]. Analysis of endothelial infiltration, co-expression analysis and expression-based survival was performed using TIMER2.0, accessed on 19 March 2022 (http://timer.cistrome.org/) [21]. Gene co-expression analysis was performed using STRING v11, accessed on 21 March 2022 (https://string-db.org/) [22]. Expression of ephrinB2 and EphB4 in different brain endothelial cells was researched using the Betsholtz dataset, accessed on 30 March 2022 (http://betsholtzlab.org/VascularSingleCells/database.html) [23,24]. Metabolic datasets were collected from Metascape, accessed on 17 March 2022 (https://metascape.org/gp/index.html#/main/step1).

### 2.2. Cell Culture

GL261 (ACC 802, DSMZ, Braunschweig, Germany) cells were cultivated in DMEM, high glucose, glutaMAX, 10% FBS and 100 U/mL penicillin-streptomycin at 37 °C and 5% CO_2_. We used the GL261 tumor cell line from C57BL/6 mice for both the orthotopic implantation and the chronic cranial window. For the conic cranial window, GL261 cells were stained with 1,1′-Dioctadecyl-3,3,3′,3′-Tetramethylindocarbocyanine Perchlorate (DiI,7,5 µg/mL growth medium) according to manufacturer’s instructions (Invitrogen). We suspended 500,000 tumor cells per ml in culture medium containing 20% Methocell (Sigma-Aldrich, Darmstadt, Germany). We distributed 100 µL of cell suspension per well to 96-well round bottom, non-adhesive, plates (Sarstedt, Nuembrecht, Germany). Round spheroids with ~500 µm diameter were selected for implantation after 48 h.

### 2.3. Mouse Breeding and Knockout Induction

This study was carried out in strict accordance with the recommendations in the Guide for the Care and Use of Laboratory Animals of the National Institutes of Health. Tamoxifen-inducible endothelial specific ephrin-B2 knockout mice (*efnb2*^i∆EC^) and *efnb2*^lox/lox^ littermates were described previously [25]. Knockout was induced in adult animals of both sexes according to the Jackson Lab guideline: using five sequential injections of 2 mg tamoxifen/mouse/day (T5648, Sigma-Aldrich, Burlington, MA, USA). Five days post final injection the animals were prepared for surgery. Control animals (*efnb2*^lox/lox^) received identical treatment.

### 2.4. Brain Histology

Immunohistochemistry was described previously [26]. Briefly, post cardial perfusion with PBS, the brain was resected and cryoprotected in 30% sucrose solution. After sinking, the brain was frozen in isopentane and stored at −80 °C. The samples were mounted in a Tissue-Tek (Sakura, Umkirch, Germany) and 20 µm coronary sections were cut with a cryostat. Sections were washed in PBS and blocked in Casein (1% *w/v* in PBS) for 30 min at room temperature (RT). The samples were incubated overnight at 8 °C with rat anti CD31 (550274, 1:50, BD Pharmingen), diluted in 0.5% Casein. Cy3-conjugated (1:200, 711-165-153, Jackson ImmunoResearch, Ely, UK) secondary antibodies were applied and incubated for 2h at RT. Nuclei were stained with 4′,6-diamidino-2-phenylindole (DAPI) and mounted for fluorescence microscopy (Immu-Mount, Thermo Fisher scientific, Dreieich, Germany).

### 2.5. Fluorescent Microscopy and Image Analysis

The slices were imaged using a Zeiss fluorescent microscope with AxioVision Software (Observer Z1, 5× EC Pln N, 5×/0.16 DIC0, resolution: 2.0 µm, 10× Pln Apo, 10×/0.45 DIC II, resolution: 0.74 µm, 20× Pln Apo, 20×/0.8 DIC II, resolution: 0.42 µm, using a HAL 100 and detectors for DAPI, GFP and DsRed and Cy5). Vascular morphology on immunohistochemical sections was based on the CellProfiler pipeline developed by Tian et al. [25]. The analysis pipeline was added to the Supplemental Material.

### 2.6. Orthotopic Intrastriatal Implantation Model

Animals of both sex, 8–11 weeks old, were obtained from the FEM (in-house animal facility). Anesthesia was induced by i.p. injection of 70 mg/kg Ketamine hydrochloride (Ketavet, Zoetis, Ilford, UK), 16mg/kg Xylazine (Rompun 2%, Bayer, Berlin, Germany) water solution. The animals were mounted in a stereo tactic frame, the skull was exposed, and hole was drilled with a 23G needle (1 mm rostral/2 mm lateral of bregma). One microliter of 2 × 10^4^/µL (PBS) tumor solution was incrementally injected over 5 min with a Hamilton syringe lowered 3 mm into the brain parenchyma. After the injection the syringe was retracted slowly over five minutes. Orthotopically implanted GL261 cells were described to form tumors with high grade glioma characteristics in immunocompetent mice [27,28,29,30]. The incision was closed and allowed to recover on a heating plate. Mice were injected with Phenoxymethylpenicillin (InfectoCilin, 5 Mega, InfectoPharm Arzneimittel und Consilium GmbH, Heppenheim, Germany) intramuscularly and the drinking water was enriched with tramadol.

### 2.7. MRI Quantification of Tumor Growth Dynamics

The MR imaging protocol has been described previously [26]. Briefly, we performed volumetric measurements of tumor mass 7-, 14- and 19-days post implantation using a 7 Tesla rodent MRI (PharmaScan 70/16 US, Bruker BioSpin MRI GmbH, Paravision 5.1, Ettlingen, Germany). A T2-weighted sequence was used showing the brain from olfactory bulb to cerebellum. Volumetric analysis was performed using ImageJ software.

### 2.8. Antiangiogenic Treatment: Sunitinib Therapy

Sunitinib (Pfizer, New York, NY, USA) dissolved in dimethyl sulfoxide (DMSO) was administered intraperitoneally (i.p.) at (80 mg/kg bodyweight/day) for 6 or 5 consecutive days as described previously [31]. Temodal therapy experiments in murine glioma suggests optimal efficacy on tumor growth when therapy is administered during the period of exponential growth [32]. Placebo animals were injected i.p. with a weight-adjusted volume of DMSO.

### 2.9. Intravital Epi-Illuminating Fluorescence Video Microscopy in a Chronic Cranial Window Model

Cranial window surgery was described previously [12,26]. Briefly, general anesthesia was induced. The animal was placed in a stereotactic frame and an approximately 5 mm diameter craniotomy in the central region of the calvaria anterior of the bregma was performed. The dura mater was removed, and the spheroid was placed subdurally onto the cortical surface. The glass window was sealed and the skin was closed with single sutures. The animal was allowed to recover for 9 days. On days 9, 12 and 14 post surgery, the newly formed, invading vasculature in the circumfluence of the tumor spheroid was imaged as described previously [26].

### 2.10. Intravital Epi-Illuminating Fluorescence Data Analysis

The CapImage 8.0 software (CAPIMAGE-Cyntel-Software-Engineering, Heidelberg, Germany) was used to analyse tumors’ marginal and central areas separately in 4–5 observations. With computer assistance microcirculation in the characteristics of the newly formed tumor vessels was quantified as total vascular density (TVD, cm/cm^2^), functional vascular density (FVD, cm/cm^2^) and perfusion index (PI = FVD/TVD).

### 2.11. Randomization and Statistical Evaluation

The animals were randomly allocated to *efnb2*^i∆EC^ or control. The experimenter was blinded for MRI-volumetry and for intravital fluorescence video microscopy. Therapy application was not blinded (technical limitation of the orange colored sunitinib solution) Statistical analysis was performed with GraphPad Prism 9 (San Diego, CA, USA). For statistical testing one-way ANOVA and two-way ANOVA with Sidak’s multiple comparison test was used. Results are presented as mean and standard deviation unless stated otherwise, *p* < 0.05 is considered significant.

## 3. Results

### 3.1. Expression Analysis of EFNB2 and EPHB4 in Physiology

**Figure 1:**
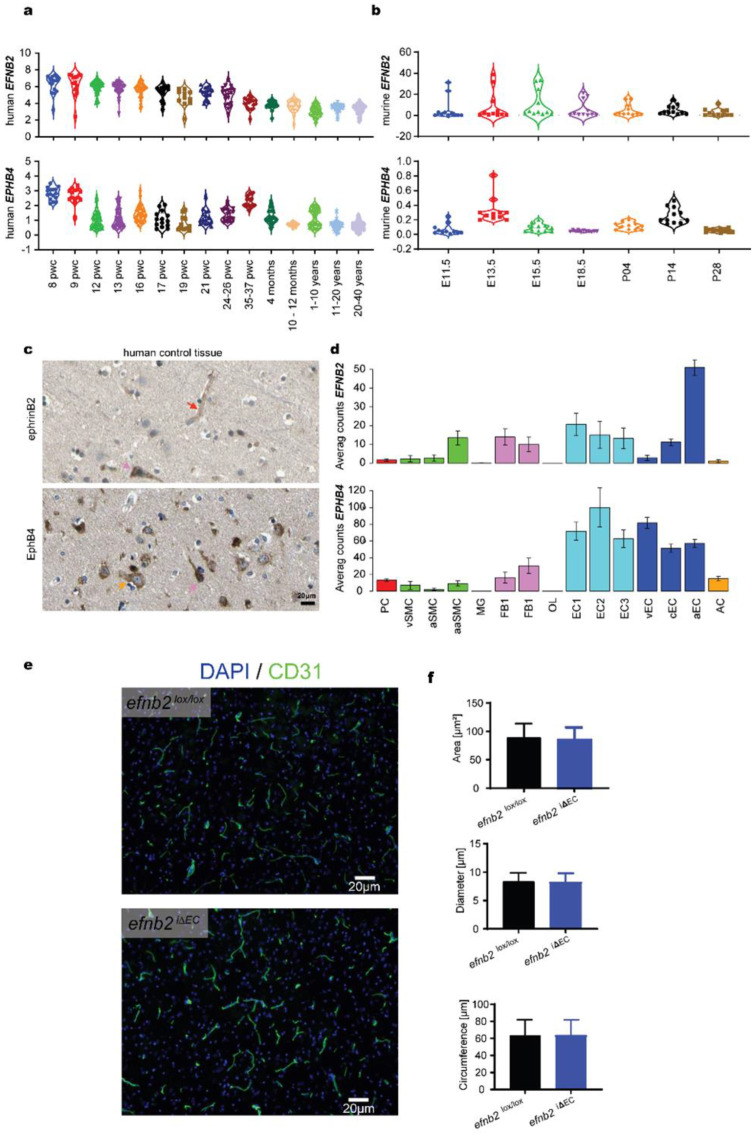
In silico analysis of ephrinB2 and EphB4 in physiological brain tissue. (**a**) Human brain tissue expression analysis form IvyGAP shows a constant decrease in EFNB2 expression during brain development. EPHB4 shows two peaks at 8 and 35–37 weeks of pregnancy (pwc) (data shown as log2 Reads Per Kilobase of transcript, per Million mapped reads (RPKM)). (**b**) Mouse brain tissue expression analysis shows a similar decreasing trend of EFNB2 expression. EPHB4 expression shows two peaks at E13.5 and P14 (data shown as expression energy). (**c**) In the healthy human brain, capillary endothelial cells (red arrow) and neurons (pink arrow) stain positive for ephrinB2 expression. EphB4 is expressed on glial cells (yellow arrow) and neurons (pink arrow). (**d**) Murine endothelial and mural cell expression profiling identify ephrinB2 expression in arterial endothelial cells. Metabolically active endothelial cells (EC1—3) show high EphB4 expression with venous endothelial cells ranking second. Histologically (**c**) and FACS-identified glial cells express EphB4 (PC—Pericytes; SMC—Smooth muscle cells; MG—Microglia; FB—Vascular fibroblast-like cells; OL—Oligodendrocytes; EC—Endothelial cells; AC—Astrocytes; v—venous; c—capillary; a—arterial; aa—arteriolar; 1,2,3- subtypes according to [23,24]). (**e**) Brain endothelial cells show no change in vascular morphology after ephrinB2 knockout. (**f**) Automated quantification of the area occupied, diameter and circumference showed no change in vascular characteristics after induced endothelial ephrinB2 knockout (*efnb2*^lox/lox^ vs. *efnb2*^i∆EC^, Student’s two-sided unpaired *t*-test).

We first analyzed the expression of EFNB2 and EPHB4 in normal brain tissue. EFNB2 expression gradually declines with age, whereas EPHB4 expression increases just before birth (35–36 pwc, Figure 1a). The murine expression in development is comparable, which makes the mouse an adequate model system to study ephrinB2 and EphB4 (Figure 1b). Histological staining of ephrinB2 showed strong expression in endothelial cells (red arrow) and neurons (pink arrow) as previously described (Figure 1c) [17,33]. EphB4 expression in human tissue was primarily located on glial cells (yellow arrow) and some neurons (pink arrow, Figure 1c). In accordance with previous findings the expression analysis of endothelial cells of different vascular compartments revealed high ERNB2 expression in arterial endothelial cells (aEC) and elevated expression of EPHB4 in venous endothelial cells (vEC, Figure 1d) [34]. Interestingly, metabolically active endothelial cells (EC1-3) show elevated levels of EPHB4. Based on this finding we performed a metabolomics search that identified only one metabolic pathway associated with EPHB4: the well-known protein tyrosine phosphate modifications induced after EPHB4 activation (Figure S1a). Tamoxifen-induced endothelial specific EFNB2 knockout in the adult mouse resulted in no changes to the vascular morphology of naïve brain tissue (Figure 1e). The area occupied by vessels, the average diameter and the circumfluence of the CD31 positive vessels remain similar (Figure 1e).

### 3.2. Expression Analysis of EFNB2 and EPHB4 in Pathology

**Figure 2:**
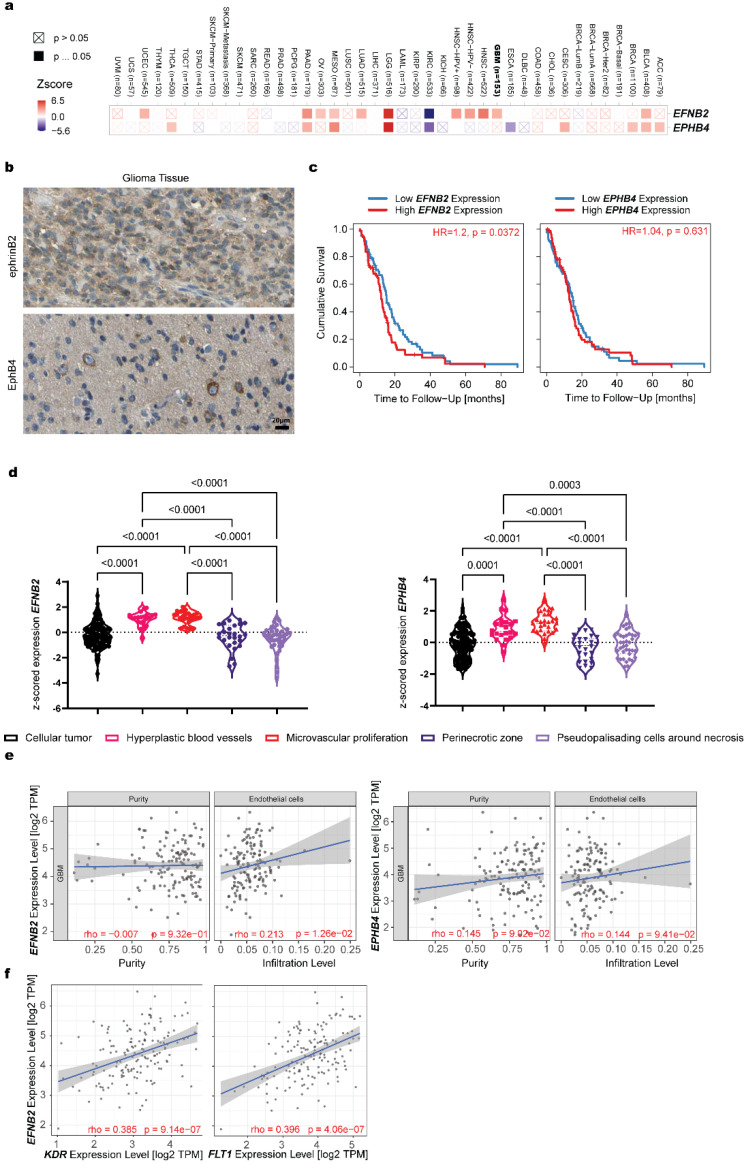
In silico analysis of ephrinB2 and EphB4 in pathological brain tissue. (**a**) EphrinB2 and the receptor EphB4 are differentially expressed depending on the tumor entity. EphrinB2 (not significant) and EphB4 (significant) expression is upregulated in glioma. The statistical significance is computed by the Wilcoxon test. (**b**) Global expression of ephrinB2 on tumor and other cells throughout the tumor was found with ephrinB2 histology. EphB4 expression in neoplasia remains localized to a small number of tumor cells. (**c**) Survival analysis shows a significant reduction in survival in patients with high ephrinB2 expression and no difference between patients with high and low EphB4 expression levels. The Cox proportional hazard model was used to evaluate the outcome significance of gene expression. (**d**) EFNB2 expression analysis based on the anatomical region shows increased expression in dens vascular areas. EPHB4 expression analysis based on the anatomical region shows increased expression in dens vascular areas (Brown-Forsythe and Welch ANOVA, with Dunnett T3 multiple comparisons test). (**e**) Increased ephrinB2 expression significantly correlated with increased infiltration of endothelial cells. On the other hand, EphB4 expression had no effect on the glioma infiltration behavior of endothelial cells. Tumor purity is a major confounding factor in this analysis since most cell types are negatively correlated with tumor purity. Therefore, we use the partial Spearman’s correlation to perform this association analysis (*p* > 0.05 = not significant, *p* < 0.05, rho < 0 = negative correlation, *p* < 0.05, rho > 0 = positive correlation). (**f**) The VEGFR2 subunits KDR and FLT1 correlate with the expression of ephrinB2. The partial Spearman’s correlation was used to perform this association analysis (*p* > 0.05 = not significant, *p* < 0.05, rho < 0 = negative correlation, *p* < 0.05, rho > 0 = positive correlation).

We screened different cancer entities for the expression of EPHB4 and EFNB2. While most cancers show an upregulation of both genes, in glioma significant upregulation was found for EPHB4 but not EFNB2 (Figure 2a, detailed plotting available in Figure S1b). Histological analysis of glioma tissue shows increased, global expression of EFNB2 and some EPHB4 expression in tumor cells (Figure 2b). Increased EFNB2 expression in glioma was shown to result in a significant reduction in patient survival. EphB4 had no effect on patient survival (Figure 2c and Figure S1c). Histological detection of ephrinB2 in glioma was very broad (Figure 2b); detailed analysis of different tumor compartments shows variable expression of ephrinB2 and EphB4. Endothelial cell rich tumor compartments, specifically, hyperplastic blood vessels and microvascular proliferative areas show increased EFNB2 and EPHB4 expression (Figure 2d). These, endothelial cell driven, EFNB2 expression changes further materialized in a different dataset, where increased endothelial cell infiltration was positively correlated with EFNB2 expression (Figure 2e). Here, EPHB4 expression showed no significant expressional changes depending on endothelial cell infiltration (Figure 2e). Based on the previous literature, ephrinB2 and VEGFR2 are known interaction partners and their dimerization is necessary for VEGFR2 internalization and angiogenic signaling [17]. We therefore examined the expression correlation of FLT1 and KDR (genes coding VEGFR2) with EFNB2 and found a positive correlation for both genes in human samples (Figure 2f).

### 3.3. Changed Glioma Growth Kinetics and Sunitinib Therapy Response after EFNB2 Knockout in Endothelial Cells

**Figure 3:**
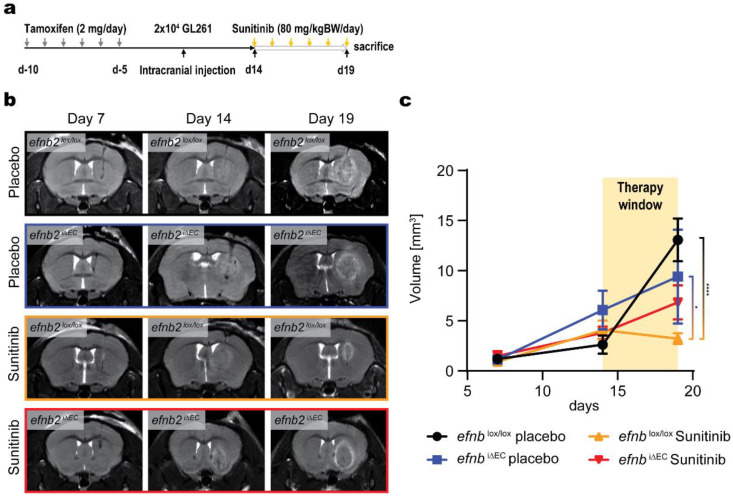
Linear in vivo growth of glioma after endothelial ephrinB2 knockout. (**a**) Experimental overview of the knockout induction and therapy experiments performed. (**b**) Coronal T2 MR images of tumor bearing mice 7, 14 and 19 days post-tumor cell injection of GL261 murine glioma cells. (**c**) T2 MR image analysis quantified a reduction in tumor growth in control animal after sunitinib therapy. Linear growth expansion of GL261 glioma was observed after endothelial specific ephrinB2 knockout (*efnb2*^i∆EC^). Exponential growth characteristics were found in control animals (*efnb2*^lox/lox^). The size between placebo control *efnb2*^lox/lox^ and sunitinib control *efnb2*^lox/lox^ (**** *p* < 0.0001) tumors and sunitinib control *efnb2*^lox/lox^ and placebo *efnb2*^i∆EC^ (* *p* = 0.0107) tumors was significant (two-way ANOVA, Sidak post hoc multiple comparisons test).

We continued to investigate endothelial expression of ephrinB2 in brain pathology. To this end, we injected GL261 murine glioma cells extrastriatally in control *efnb2*^lox/lox^ and *efnb2*^i∆EC^ animals and performed T2 weighted MRI 7, 14 and 19 days after tumor cell injection in combination with antiangiogenic therapy (experimental overview Figure 3a). We observed different tumor growth kinetics in *efnb2*^i∆EC^ animals (Figure 3b). Growth was found to be linear, independent of the treatment group in *efnb2*^i∆EC^ animals. In control animals, glioma growth was exponential in the placebo group compared to the sunitinib group in which this growth kinetic was interrupted with therapy induction. (Figure 3c). A significant reduction in tumor growth was found in the sunitinib *efnb2*^lox/lox^ group.

### 3.4. Functional Characterization of Glioma Angiogenesis in efnb2^i∆EC^ Animals with Sunitinib Therapy

**Figure 4:**
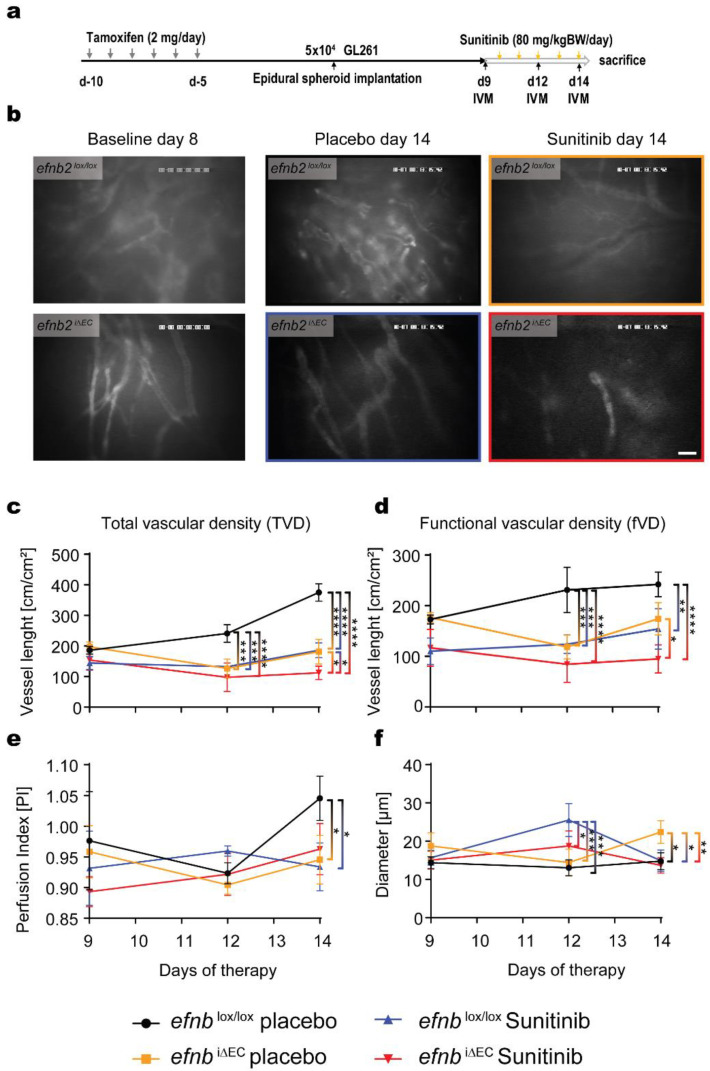
Intravital fluorescence microscopy visualization of *efnb2*^i∆EC^ vasculature in glioma. (**a**) Experimental design and procedure of the Intravital microscopy (IVM) experiments. (**b**) Exemplary images of vascular architecture in control and *efnb2*^i∆EC^ animals 14 days after tumor cell implantation with placebo or sunitinib therapy, respectively (scale bar: 60 µm). (**c**) Total vascular density (tVD) quantified in control and *efnb2*^i∆EC^ animals revealed a significant difference in placebo control tumors 12 and 14 days (*** *p* = 0.0005 and **** *p* < 0.0001) after implantation. Sunitinib therapy showed a significant effect in control animals at both timepoints (*** *p* = 0.0003 and **** *p* < 0.0001). Sunitinib therapy in *efnb2*^i∆EC^ animals significantly reduced TVD at day 14 compared to sunitinib control *efnb2*^lox/lox^ and placebo *efnb2*^i∆EC^ animals (* *p* = 0.036 and * *p* = 0.0229). Reduced TVD in placebo control animals was found on day 12 and 14 after tumor implantation (*** *p* < 0.0001 and **** *p* < 0.0001). (**d**) Functional vascular density (fVD) quantified showed a significant therapeutic difference in control tumors after 12 days (*** *p* = 0.0005) and recovery after 14 days. Endothelial ephrinB2 depletion (*efnb2*^i∆EC^) reduced fVD 12 and 14 days after surgery (*** *p* = 0.001 and ** *p* = 0.0072) with an add on effect observed under sunitinib therapy (day 12: **** *p* < 0.0001, day 14 **** *p* < 0.0001). EphrinB2 depletion in endothelial cells (*efnb2*^i∆EC^) showed an additional fVD reduction 14 days after tumor implantation (* *p* = 0.0175). (**e**) Blood perfusion index (PI) shows no difference until 5 days of therapy where control placebo perfusion is significantly higher compared to the *efnb2*^lox/lox^ sunitinib group (* *p* = 0.0396) and *efnb2*^i∆EC^ placebo animals (* *p* = 0.0172). (**f**) EphrinB2 knockout animals (*efnb2*^i∆EC^ placebo) show a significant increase in diameter 12 days after tumor cell implantation (*efnb2*^lox/lox^ placebo: *** *p* = 0.0001, *efnb2*^lox/lox^ sunitinib: *** *p* = 0.0005 and *efnb2*^i∆EC^ sunitinib: * *p* = 0.0484). Two days later this increase is normalized and only the *efnb2*^lox/lox^ sunitinib group showed a significant increase in diameter (*efnb2*^lox/lox^ placebo: * *p* = 0.0212, *efnb2*^i∆EC^ placebo: * *p* = 0.0240, *efnb2*^i∆EC^ sunitinib: ** *p* = 0.0067, (**c**–**f**): two-way ANAOVA analysis, Sidak’s multiple comparisons test used for all statistics).

Nine days after frontal cortical placement of a GL261 tumor spheroid, the angiogenic phenotype was investigated using intravital epifluorescence microscopy. Similar to the intra striatal tumor injection experiments, control and *efnb2*^i∆EC^ mice received antiangiogenic and placebo therapy. Considering the increased number of cells used for inoculation in these animals, we started the therapy 9 days post implantation. Baseline imaging was performed at day 9, and consecutive observations followed at day 12 and 14 (experimental protocol Figure 4a). We visualized changes in vascular density, perfusion, blood velocity, blood flow and diameter (Figure 4b and Figure S2). Apart from the changes in vascular diameter, *efnb2*^i∆EC^ resembled most of the vascular phenotypes observed in the control antiangiogenic therapy group (Figure 4c–e). Moreover, sunitinib had an add on effect in the ephrinB2 knockout animals in the total vascular density (TVD) measurements (Figure 4c). Vascular morphology (diameter) revealed interesting dynamics with placebo-treated *efnb2*^i∆EC^ animals showing a significant increase in diameter 12 days after spheroid implantation and a further reduction 2 days after compared to the relatively unchanged average diameter in *efnb2*^i∆EC^ animals and placebo control animals (Figure 4f). sunitinib therapy in controls resembled the well-characterized resistance formation phenotype with large vessel diameters 14 days post-therapy, after an initial reduction in blood vessels 3 days post-therapy (Figure 4f).

## 4. Discussion

Based on TCGA and other public datasets, we identified ephrinB2 as a driver of gliomagenesis. In-depth analysis revealed invading endothelial cells to express high amounts of ephrinB2. These findings align well with the established vascular phenotype in glioma induced by VEGFA secretion and the observed vascular VEGFR2 regulation function of ephrinB2 [17]. We further strengthened this argument by correlating the expression of the two VEGFR2 subunit genes FLT1 and KDR with the expression of EFNB2 in Glioma.

In vitro analysis of ephrinB2 in endothelial cells found binding to CD31 [35]. EphB4 positive endothelial cells segregated to small compartments when co-cultured with ephrinB2 positive cells [36]. Hence, the authors concluded that this pathway restricts intermingling of arterial cells expressing EFNB2 with venous cells that express EPHB4 in vasculogenesis. Previous in vivo reports identified vascular ephrinB2 to be essential in embryonic development and modulation of pericyte recruitment [37,38]. However, the brain vasculature was unaffected in the adult animal after Tamoxifen induced ephrinB2 knockout as previously described [39]. We therefore continued our investigation regarding endothelial ephrinB2 in glioma in combination with antiangiogenic therapy. We observed that endothelial ephrinB2 knockdown changed the growth behavior of gliomas and reduced susceptibility to antiangiogenic therapy through vascular morphology alterations. We hypothesize that the interference in endothelial VEGFR2 signaling induces hypoxic conditions similar to the therapeutic blockade of VEGF [40,41]. In consequence, other proangiogenic pathways are activated [42,43]. These signaling pathways are not as potent in providing nutrients so that tumor growth cannot occur exponentially [44]. Hence, slower, more physiological growth is observed.

Intravital microscopy studies confirm this hypothesis. The alternations in microvascular flow in *efnb2*^i∆EC^ animals reconstitute the changes observed with the antiangiogenic agent sunitinib. Sunitinib was favored over Bevacizumab in this study based on the reported lack of immune neutralization of murine VEGFA by Bevacizumab [45]. Sunitinib reproduced the well reported antiangiogenic effects in murine glioma [31,46]. However, introducing this therapy in glioma spheroid bearing *efnb2*^i∆EC^ animals had little to no additional effect even though multiple off-target effects of sunitinib have been reported [47]. We consider two hypotheses explaining this observation; first, sunitinib exerts its therapeutic potency primarily by inhibition of VEGFR2 in this spheroid model of murine glioma. Second, ephrinB2 has more diverse molecular membrane interaction partners than previously anticipated and the knockdown affects multiple downstream pathways [40,48]. Both are subject to further investigation. In this study we lack molecular downstream analysis to verify the exact downstream convergence of ephrinB2 and VEGFR2 signaling; another limitation is the lack of clinical translation through therapeutic interventions with ephrinB2–EphB4, a field of active research. Most ephrinB2–EphB4 therapeutics today target the EphB4 receptor [40]. Only the preclinical agent EphB4-FC targets the extracellular domain of ephrinB2 with unknown downstream consequences in vivo. Internalization of ephrinB2-FC/EphB4-FC has been reported in vitro and it is unknown if this results in the activation of downstream effectors or inhibition of the ephrinB2-EphB4 signaling [49,50,51]. Recently, the promising bimodular inhibitor BIDEN-AP based on TNYL-RAW has been developed that inhibits both ephrinB2 forward and EphB4 reverse signaling [52].

However, ephrinB2–EphB4 signaling is complex: it can lead to tumor progression and tumor arrest/reduction, depending on the cancer entity, ligand expression, receptor expression, internalization status, forward–reverse signaling balance, the microenvironment the tumor grows in and many other unidentified aspects. We extensively reviewed this phenomenon in the neurooncological context, concluding that more molecular tools must be developed to address the Janus-faced nature of ephrinB2–EphB4 signaling in cancer [40].

In summary, our findings indicate that ephrinB2 and VEGFA converge on similar downstream pathways in endothelial cells in vivo. Interference with either molecule resulted in comparable pathological and vascular changes.

## Data Availability

The data that support the findings of this study are available from the corresponding author upon reasonable request.

## Supplementary Materials

The following supporting information can be downloaded at: https://www.mdpi.com/article/10.3390/life12050691/s1, Figure S1: (a) Metabolic pathway analysis of EphB4 tyrosine receptor kinase shows involvement in general cytosol metabolism, (b) Detailed expression analysis of ephrinB2 and EphB4 in different tumor types (red) and controls (blue), (c) Survival probability depending on EFNB2 expression obtained from the Protein Atlas Dataset; Figure S2: (a) Blood velocity was unimpaired in the different groups, (b) Blood flow was unimpaired in the different groups.
