# Supplementary material for: Endothelial EphrinB2 Regulates Sunitinib Therapy Response in Murine Glioma
Source: Life (Basel). 2022 May 6;12(5):691. doi: 10.3390/life12050691 (PMC9146972; doi:10.3390/life12050691)
Supplement: Supplementary file 1 [file life-12-00691-s001.zip › SupplementalFigure.pdf]

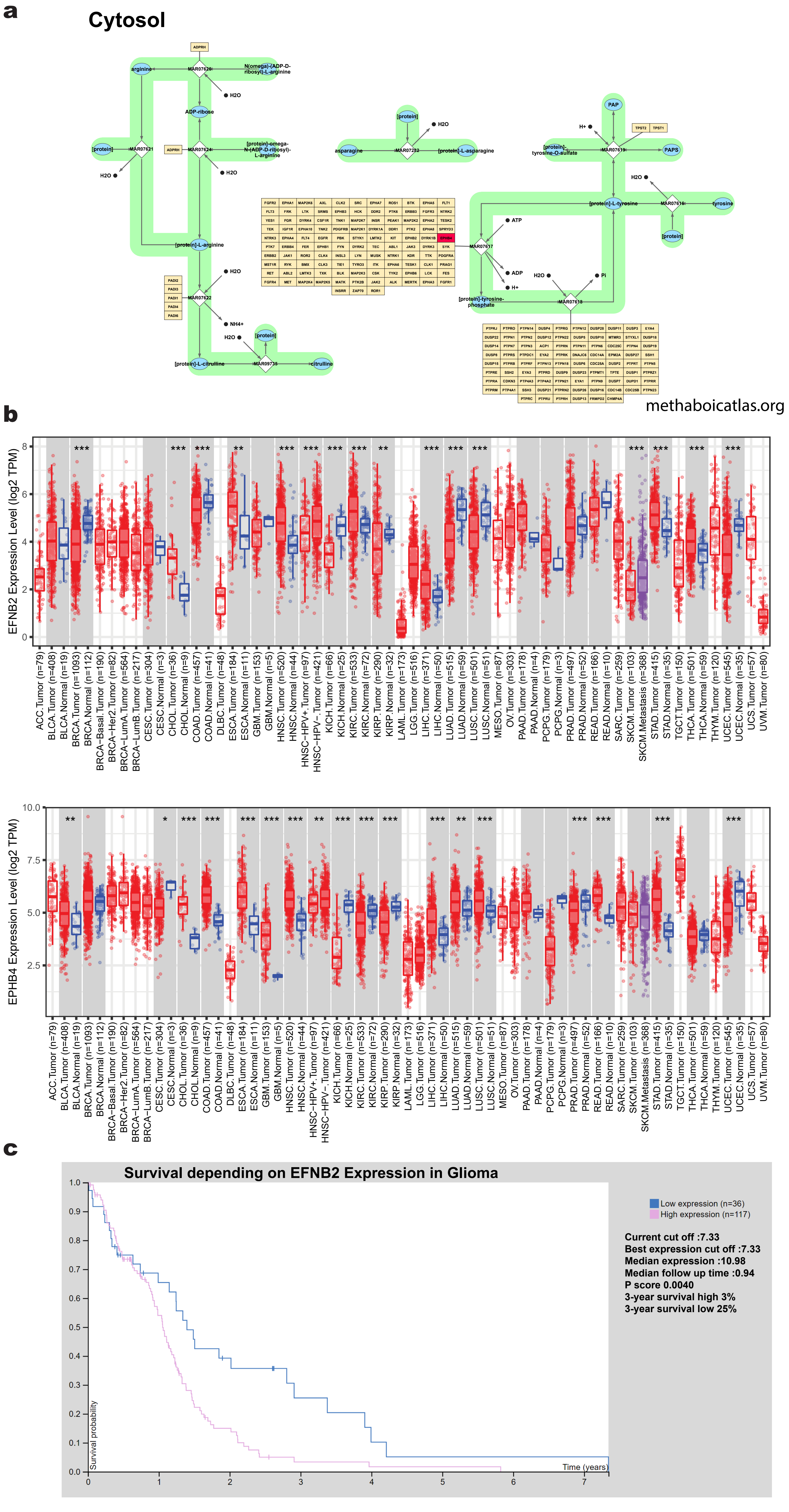

Figure S1:
(a) Metabolic pathway analysis of EphB4 tyrosine receptor kinase shows involvement in general cytosol metabolism, (b) Detailed expression analysis of ephrinB2 and EphB4 in different tumor types (red) and controls (blue), (c) Survival probability depending on EFNB2 expression obtained from the Protein Atlas Dataset

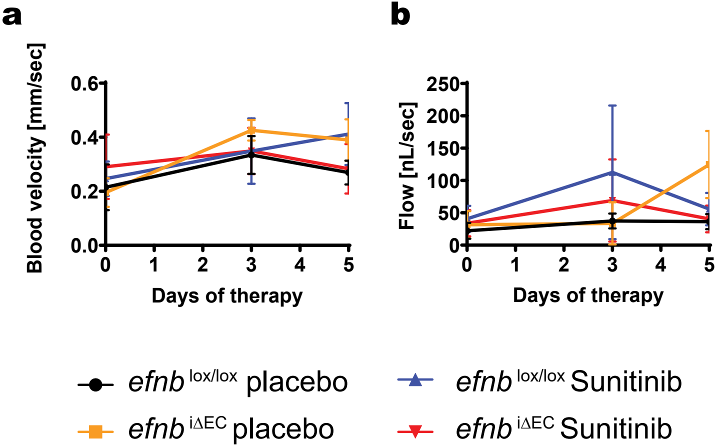

Figure S2:
(a) Blood velocity was unimpaired in the different groups, (b) Blood flow was unimpaired in the different groups.
